# Gold Clusters Attenuate Inflammation in Rat Mesangial Cells via Inhibiting the Activation of NF-κB Pathway

**DOI:** 10.3390/nano10040712

**Published:** 2020-04-10

**Authors:** Jinling Yuan, Kaixiao Hou, Yawen Yao, Zhongying Du, Cao Lu, Qing Yuan, Xueyun Gao

**Affiliations:** Department of Chemistry and Chemical Engineering, Beijing University of Technology, Beijing 100124, China; jinlingy316@163.com (J.Y.); yaoyw@emails.bjut.edu.cn (Y.Y.);

**Keywords:** sepsis-induced acute kidney injury, inflammation, gold clusters, mesangial cell, NF-κB pathway

## Abstract

Sepsis-induced acute kidney injury (AKI) with high incidence and mortality rates remains a great challenge in the clinic; thus, novel therapies need to be developed urgently. This complication is associated with an overwhelming systemic inflammatory response. The aim of this study was to evaluate the potential effects and possible mechanisms of gold clusters on septic AKI in vitro. Rat mesangial HBZY-1 cells were treated with peptide-templated gold clusters under lipopolysaccharide (LPS) stimulation. The LPS-induced expression of pro-inflammatory cytokines was measured, including tumor necrosis factor alpha (TNF-α), interleukin-1 beta (IL-1β) and interleukin-6 (IL-6). Our data showed that the LPS-induced transcription and secretion of these cytokines were suppressed by pretreatment of gold clusters in a dose-dependent manner. Cyclooxygenase-2 (COX-2) and inducible nitric oxide synthase (iNOS) also play key roles in septic AKI and both of them are induced upon LPS-stimulation in mesangial cells. Our results further showed that pretreatment with gold clusters dramatically inhibited the LPS-stimulated transcription and expression of COX2 and iNOS, and the subsequent prostaglandin E2 (PGE2) and nitric oxide (NO) production in HBZY-1 cells. Since these factors are involved in the NF-κB pathway upon LPS stimulation, the potential roles of gold clusters on the NF-κB pathway were further determined. We found that LPS-induced NF-κB activation was suppressed in gold clusters-pretreated HBZY-1 cells. These results demonstrated that gold clusters can attenuate LPS-induced inflammation in mesangial cells, probably via inhibiting the activation of the NF-κB pathway, suggesting a potential therapeutic approach for septic AKI.

## 1. Introduction

Sepsis initiated by invasive infection is a leading cause of death in intensive care units (ICU), which is characterized by a systemic inflammatory response [1,2]. The mortality rate of sepsis is up to 60%, and about 14,000 people die of its complications worldwide every day [3] The high mortality rate of sepsis is usually directly caused by multiple organ failure [4]. Sepsis can affect multiple organs via the overwhelming induction of pro-inflammatory cytokines [1]. Acute kidney injury (AKI) is a common and serious complication of sepsis [5,6]. It has been reported that about half of the patients with sepsis suffer from AKI, which is a devastating disease characterized by sudden impairment of the renal function [7]. Patients with septic AKI have more severe abnormalities in acute physiology, thus increasing the risk of death [8,9]. To date, although a lot of efforts have been made on the research of anti-sepsis drugs, the treatment for sepsis-caused AKI is still unsatisfactory [10]. Therefore, it is crucial to develop novel therapeutic approaches due to the huge health threats of AKI.

Gold clusters are a novel type of gold nanomaterial with an ultra-small size (usually smaller than 2 nm) and well-defined molecular structure, making them possess various unique physicochemical and biomedical properties that are not seen in the corresponding bigger nanomaterials or bulk materials [11]. In particular, gold clusters prepared with peptides have recently attracted extensive attention in the field of biomedicine, due to their excellent biocompatibility and intrinsic biomedical properties [11,12]. We have prepared two peptide-templated gold clusters by using glutathione and Sv peptide respectively, named Au_29_SG_27_ and Au_25_Sv_9_ [13,14]. Our previous studies demonstrated that these two gold clusters exhibited anti-inflammation activities in murine macrophage cells (RAW264.7) and microglia cells (BV-2), two immune cells that mediate the inflammatory response in the peripheral and central nervous system, respectively [13,15,16]. However, the effects of gold clusters on septic AKI and its related molecular mechanism remain unclear. 

**Scheme 1:**
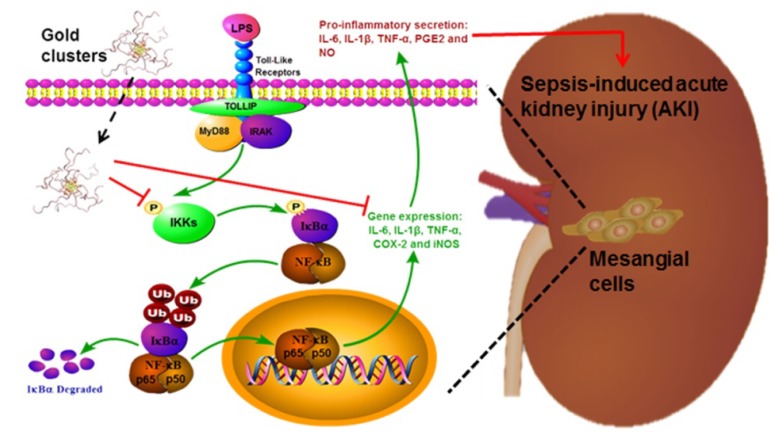
Gold clusters suppress LPS-induced inflammatory responses in Rat mesangial cells via inhibiting the activation of the NF-κB pathway. LPS: lipopolysaccharide; NF-κB: nuclear factor kappa B; IκBα: NF-κB inhibitor alpha; p-IκBα: phosphorylated IκBα; IKKs: IκB kinases; IL-6: interleukin-6; IL-1β: interleukin-1 beta; TNF-α: tumor necrosis factor alpha; COX-2: Cyclooxygenase-2; iNOS: inducible nitric oxide synthase; PGE2: prostaglandin E2; and NO: nitric oxide.

Therefore, in this study, we aim to clarify the potential effects of gold clusters in LPS-stimulated rat glomerular mesangial cells (HBZY-1). Since the LPS-induced inflammatory response in mesangial cells is mainly triggered by the NF-κB pathway, the effects of gold clusters on NF-κB activation were also examined in LPS-treated HBZY-1 cells. We found that gold clusters could effectively inhibit the LPS-stimulated overexpression of pro-inflammatory factors in HBZY-1 cells, including TNF-a, IL-1β, IL-6, iNOS and COX-2, probably via suppressing the activation of the NF-κB pathway (Scheme 1).

## 2. Materials and Methods

### 2.1. Preparation of Gold Clusters

#### 2.1.1. Preparation of Au_25_Sv_9_ Gold Cluster

The gold cluster was prepared according to our previously published methods [14,15]. 2.5 mg of the Sv peptide (H_2_N-Cys-Cys-Tyr-Gly-Gly-Pro-Lys-Lys-Lys-Arg-Lys-Val-Gly-COOH) was dissolved in 1.505 mL ultra-pure water, slowly adding HAuCl_4_ (25 mM, 70 μL) under vigorous stirring. After 2 min, NaOH (0.5 M, 175 μL) was dripped into the above solution, stirring for another 3 min. The whole reaction was carried out in the dark at room temperature. Then, the sample was placed in darkness for 3 days. An ultrafiltration tube (Molecular weight cut off: 3 kDa) was used for further concentration and purification. An aliquot of the product was taken to be quantified by inductively coupled plasma mass spectrometry (ICP-MS), and the rest was stored at 4 °C in the dark.

#### 2.1.2. Preparation of Au_29_SG_27_ Gold Clusters

The gold cluster was also prepared according to our previously published methods [13,17]. 9.2 mg glutathione (GSH: γ-Glu-Cys-Gly) was dissolved in 1.2 mL ultra-pure water. At room temperature, HAuCl_4_ (25 mM, 80 mL) was slowly introduced into the peptide solution. After magnetic stirring for 10 min, the reaction was carried out in a 70 °C water bath for 12 h. The sample was placed in darkness for another 12 h. After that, the as-prepared gold cluster was purified by adding three volumes of ethanol and was centrifuged at 10,000 rpm for 15 min. The obtained precipitate was washed three times with 75% ethanol. The resulting precipitate was dissolved with 0.25 M of NaOH and adjusted to PH = 7. Finally, to purify the product to remove free peptides we used the ultrafiltration tube (MWCO: 3 kDa). Similar to Au_25_Sv_9_ cluster products, an aliquot of Au_29_SG_27_ clusters was taken to be measured by ICP-MS, and the rest was stored at 4 °C in the dark.

### 2.2. Characterization of Gold Clusters

The luminescence of purified gold clusters was observed in the dark by using a portable ultraviolet analyzer (ZF-5, Nanjing, China). The fluorescence spectrophotometer (Shimadzu RF-5301, Kyoto, Japan) was used to acquire the fluorescence spectra of the as-synthesized gold clusters. Ultraviolet-visible absorption spectra of gold clusters were acquired by a spectrophotometer (Shimadzu UV-1800, Kyoto, Japan). A TENCAI F20 high-resolution transmission electron microscopy (HRTEM, FEI, Hillsboro, OR, USA) was used to observe the synthetic gold clusters, at 200 kV accelerating voltage.

### 2.3. Cell Culture and Treatment

A rat HBZY-1 glomerular mesangial cell line obtained from the Cell Bank of Chinese Academy of Sciences (Beijing, China) was cultured in Dulbecco’s modified Eagle medium (DMEM; Gibco, Grand Island, NY, USA) supplemented with 10% fetal bovine serum (FBS; Gibco, Logan, UT, USA) and penicillin/streptomycin (100 μg/mL) at 37 °C in 5% CO_2_-humidified atmosphere. HBZY-1 cells were plated in six-well plates at a density of 4 × 10^5^ cells per well and allowed to grow until reaching a confluence of 75%–85%. Then, the cells were pretreated with or without gold clusters for 30 min. Subsequently, the cells were stimulated with 0.1 μg/mL LPS (Sigma-Aldrich, Saint Louis, MO, USA) for indicated times.

### 2.4. Cells Counting Kit-8 Assay for Cell Viability

HBZY-1 cells were seeded into 96-well plates at a density of 5 × 10^3^ cells per well and were incubated at 37 °C for 24 h. Then, the cells were treated with 5, 10, 20, 50 and 100 μM gold clusters for 24 h. Next, 10 μL of Cell Counting Kit (CCK-8) reagent (Dojindo Laboratories, Kumamoto, Japan) diluted with 100 μL medium was added to each well, and incubated for 30 min; the absorbance at 450 nm was measured using a microplate reader (Molecular Devices, San Jose, CA, USA).

### 2.5. Measurement of TNF-α, IL-1β, IL-6 and PGE2

The supernatant of the HBZY-1 cells was used to detect the production levels of the inflammatory factors, including tumor necrosis factor alpha (TNF-α), interleukin-1 beta (IL-1β), IL-6 and PGE2. These factors were quantified by the enzyme-linked immunosorbent assay (ELISA) kits (J&L biological, Shanghai, China) according to the instructions and expressed as pictograms (pg) per milliliter (mL) of cell supernatant.

### 2.6. Measurement of NO Release

The LPS-induced production of total NO in HBZY-1 cells was detected. 60 μL of cell lysate was measured with a Total Nitric Oxide Assay Kit according to the manufacturer’s instructions (Beyotime Biotechnology, Shanghai, China). The absorbance at 540 nm was measured using a microplate reader (Molecular Devices, San Jose, CA, USA).

### 2.7. Reverse Transcription PCR (RT-PCR) and Real-Time Quantitative PCR (RT-qPCR)

After LPS stimulation (0.1 μg/mL) for 12 h, the total mRNA of HBZY-1 cells was extracted by using the RNeasy Kit (50) Plus Mini Qiagen Kit (74134, Qiagen) according to the instructions. The cDNA was made by the reverse transcription kit (Trans Script^®^ All-in-One First-Strand cDNA Synthesis Super Mix for qPCR, TransGen Biotech, Beijing, China) in a 20 μL reaction system. The amplification of each target gene was carried out in a 25 μL reaction system by using the Perfect Start^TM^ Taq DNA Polymerase kit (TransGen Biotech, Beijing, China) and observed through agarose gel electrophoresis. The real-time quantitative PCR was performed by using the LightCycler^®^ 96 Real-Time PCR System (Roche, Basel, Switzerland) and *PerfectStart*^TM^ Green qPCR SuperMix (TransGen Biotech, Beijing, China). The results were quantitatively analyzed by a 2^−ΔΔCt^ method. GAPDH was used as the internal control, and the primers sequences are shown in Table 1.

### 2.8. Western Blotting

Nuclear and cytoplasmic proteins were extracted from HBZY-1 cells using the Nuclear and Cytoplasmic Extraction Reagent Kit (Beyotime Biotechnology, Shanghai, China). The concentration of each protein sample was determined by BCA reagent (Beyotime Biotechnology, China). The total protein was separated by 12% SDS-PAGE and transferred to PVDF membranes. After blocking with 5% BSA (w/v) for 1 h at room temperature, the membranes were incubated with primary antibodies for COX-2, iNOS, nuclear factor kappa B (NF-κB), inhibitor alpha (IκBα), phosphorylated IκBα (p-IκBα), IκB kinase beta (IKKβ), phosphorylated IKKβ (p-IKKβ) and p65, respectively, overnight at 4 °C and then incubated with secondary antibodies at room temperature for 1 h. Immunoreactive bands were visualized by ECL kit (Millipore), and the densitometry was analyzed by ImageJ software (NIH, Bethesda, MD, USA).

### 2.9. Statistical Analysis

The statistical analysis was performed using SPSS software version 19.0 (Chicago, IL, USA). Statistical differences were assessed by a one-way ANOVA comparison, and P < 0.05 was considered to be statistically significant. Data are represented as the mean ± standard deviation (SD). 

## 3. Results

### 3.1. Characterization of Synthesized Gold Clusters

**Figure 1:**
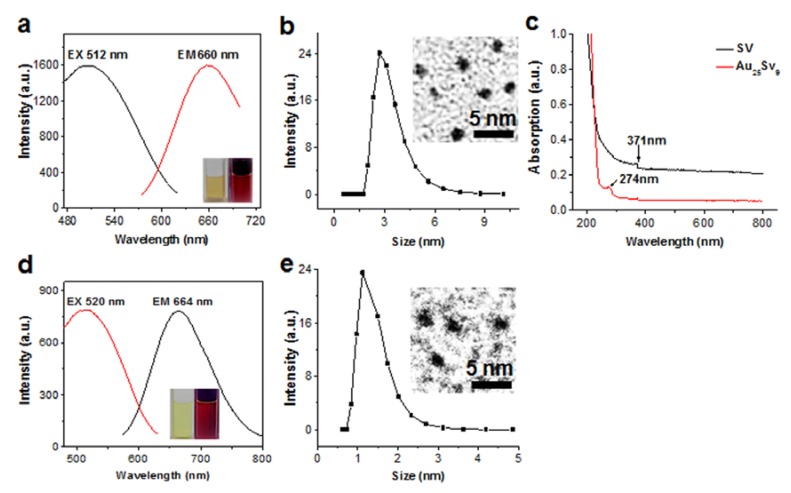
Characterization of the as-synthesized gold clusters. (**a**) The fluorescence excitation (512 nm) and emission spectra (660 nm) of Au_25_Sv_9_. Inset: digital images of the Au_25_Sv_9_ under sunlight (left) and ultraviolet light (right). (**b**) Size distribution of Au_25_Sv_9_ measured by dynamic light scattering (DLS). Inset shows a representative transmission electron microscopy (TEM) image of Au_25_Sv_9_, scale bar 5 nm. (**c**) UV-Vis (Ultraviolet-visible absorption spectra) analysis of the Sv peptide and Au_25_Sv_9_ clusters. (**d**) The fluorescence excitation (520 nm) and emission spectra (664 nm) of Au_25_SG_27_. Inset: digital images of the Au_25_SG_27_ under sunlight (left) and ultraviolet light (right). (**e**) Size distribution of Au_25_SG_27_ measured by DLS. Inset shows a representative TEM image of Au_29_SG_27_, scale bar 5 nm.

In this study, two well-defined peptide-templated gold clusters were synthesized with Sv peptide (Au_25_Sv_9_) or GSH (Au_29_SG_27_) respectively, according to our previously published methods [13,14,15,17]. Both gold clusters showed a good water solubility and excellent photoluminescence properties. The solution containing Au_25_Sv_9_ displays as brown under sunlight and emits a strong red fluorescence under UV light excitation (inset of Figure 1a). According to the spectral analysis, the peaks of the excitation and emission spectra of Au_25_Sv_9_ were located at 512 nm (black curve) and 660 nm (red curve), respectively (Figure 1a). The hydrodynamic size of Au_25_Sv_9_ was 3.43 ± 1.04 nm, determined by dynamic light scattering (DLS) detection (Figure 1b). The TEM image showed that the synthesized gold cluster has a fairly good water dispersion and that the core size is around 2 nm (inset of Figure 1b). The UV-Vis spectrum analysis showed that Au_25_Sv_9_ had a characteristic absorption peak at 274 nm and that that of Sv peptide was at 371 nm, indicating the formation of gold clusters (Figure 1c).

The solution of Au_29_SG_27_ displays as light yellow under sunlight and also emits a strong red fluorescence under UV light (inset of Figure 1d). The spectral analysis showed that the peak of excitation and emission of Au_29_SG_27_ was located at 520 nm (red curve) and 664 nm (black curve), respectively (Figure 1d). DLS detection showed that the hydrodynamic size of Au_29_SG_27_ was 1.51 ± 0.38 nm (Figure 1e). The TEM image showed that the synthesized gold cluster also has a good water dispersion and that the core size is around 2 nm (inset of Figure 1e).

### 3.2. Effects of Au_25_Sv_9_ on HBZY-1 cell Viability and LPS-induced Inflammatory Cytokines Expression

**Figure 2:**
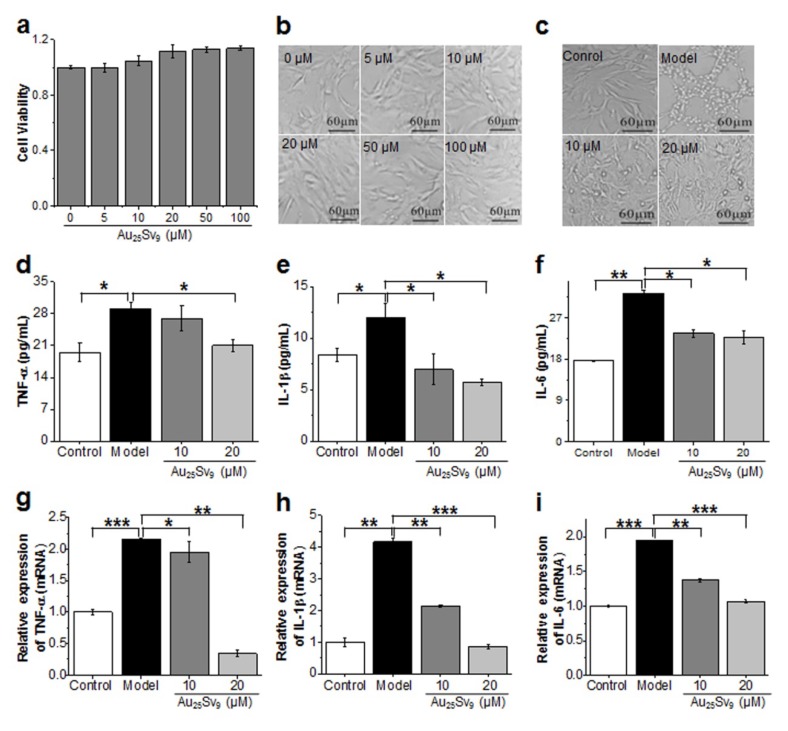
Effects of Au_25_Sv_9_ on HBZY-1 cell viability and inflammatory cytokine expression. (**a**) Cell viability of HBZY-1 cells after incubation with different doses of Au_25_Sv_9_ for 24 h. The data was presented as the mean of triplicate experiments. (**b**) Cell morphology of HBZY-1 cells after incubation with different doses of Au_25_Sv_9_ for 24 h. The representative images were shown. (**c**) Cell morphological changes of HBZY-1 cells after 0.1 μg/mL LPS (lipopolysaccharide) stimulation with or without Au_25_Sv_9_ pretreatment for 24 h. The representative images were shown. (**d**–**f**) The effect of Au_25_Sv_9_ on LPS-induced TNF-α (tumor necrosis factor alpha), IL-1β (interleukin-1 beta) and IL-6 (interleukin-6) secretion in the supernatant of HBZY-1 cells after 24 h incubation. Results were expressed as the mean of three repeated experiments ± standard deviation (SD), * P < 0.05, ** P < 0.01. (**g**–**i**) The effect of Au_25_Sv_9_ on the LPS-induced transcription of TNF-α, IL-1β and IL-6 in HBZY-1 cells after 12 h incubation were detected by RT-qPCR (real-time quantitative PCR). GAPDH was used to normalize the data. Data are presented as the mean of three repeated experiments ± SD, * P < 0.05, ** P < 0.01, *** P < 0.001.

The LPS-stimulated rat mesangial cell line HBZY-1 is a well-established septic AKI model in vitro [18,19]. The cytotoxicity of Au_25_Sv_9_ gold clusters on HBZY-1 cells was first evaluated by CCK-8 assay. The viability of HBZY-1 cells was measured after incubation with various concentrations of gold clusters, from 0 to 100 μM, for 24 h. Our results indicated the gold clusters did not cause any obvious cytotoxicity to HBZY-1 cells, even at the concentration of 100 μM (Figure 2a). Cell morphology changes were not observed after incubation with Au_25_Sv_9_, indicating a good biocompatibility of this gold cluster (Figure 2b). After LPS stimulation, the HBZY-1 cells become shrunk, indicating an inflammatory activation (Figure 2c). However, morphological changes induced by LPS-stimulation can be rescued significantly by pretreatment with Au_25_Sv_9_ in a dose-dependent manner, suggesting a potential anti-inflammatory activity of the gold clusters (Figure 2c). 

Moreover, the levels of IL-6, IL-1β and TNF-α were dramatically increased in the supernatant of HBZY-1 cells after LPS-stimulation for 24 h, demonstrating that the inflammatory model was successfully established. Treatment with Au_25_Sv_9_ could effectively suppress the secretion of these pro-inflammatory factors in a dose-dependent manner (Figure 2d–f). Consequently, effects of Au_25_Sv_9_ on the LPS-induced transcription of these factors were detected by RT-qPCR. As shown in Figure 2, LPS-stimulation increased the mRNA levels of IL-6, IL-1β and TNF-α in HBZY-1 cells dramatically, while pretreatment with Au_25_Sv_9_ was able to suppress this induction effectively (Figure 2g–i). 

### 3.3. Au_25_Sv_9_ Inhibited COX-2 and iNOS Expression in LPS-Stimulated HBZY-1 Cells

**Figure 3:**
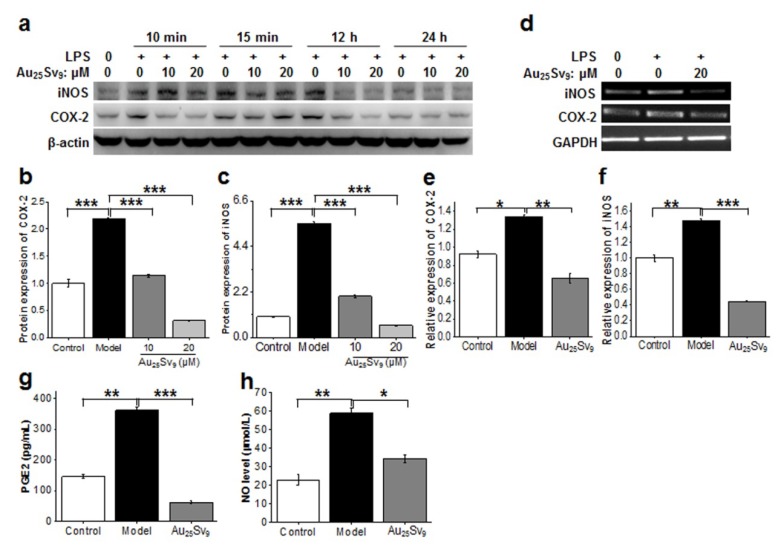
The effect of Au_25_Sv_9_ on COX-2 (Cyclooxygenase-2) and iNOS (inducible nitric oxide synthase) expression and PGE2 (prostaglandin E2) and NO (nitric oxide) production in LPS (lipopolysaccharide)-stimulated HBZY-1 cells. (**a**) Western blotting detection of the expression of COX-2 and iNOS at different time points. β-actin was used as the loading control. (**b**) The densitometry analysis of COX-2 immunoreactive bands at 12 h; β-actin was used to normalize the data. Data was expressed as the mean ± standard deviation (n = 3). (**c**) The densitometry analysis of iNOS immunoreactive bands at 12 h. β-actin was used to normalize the data. Data are presented as the mean ± standard deviation (n = 3). (**d**–**f**) The effect of Au_25_Sv_9_ on the LPS-induced transcription of COX-2 and iNOS in HBZY-1 cells after 12 h incubation. GAPDH was used as the internal control and was used to normalize the data. (**d**) Representative images of RT-PCR (Reverse transcription PCR) and (**e**,**f**) the quantitative analysis of RT-qPCR (real-time quantitative PCR) were shown. Each data was expressed as the mean ± standard deviation (n = 3). (**g**,**h**) The effect of Au_25_Sv_9_ on the LPS-induced production of PGE2 and NO in the supernatant of HBZY-1 cells after 24 h incubation. Data are presented as the mean ± standard deviation (n = 3). * P < 0.05, ** P < 0.01, *** P < 0.001.

Considering the key roles of COX-2 and iNOS in renal inflammatory response [20,21], we measured the expression of COX-2 and iNOS in HBZY-1 cells with or without Au_25_Sv_9_ treatment, after stimulation by LPS. Western blotting results indicated that the protein expression of COX-2 and iNOS was dramatically upregulated in HBZY-1 cells upon LPS stimulation, while Au_25_Sv_9_ treatment suppressed their overexpression at different time points (Figure 3a–c). As shown in Figure 3, Au_25_Sv_9_ also significantly inhibited LPS-induced COX-2 and iNOS expression at the mRNA level (Figure 3d–f). The inflammatory mediators PGE2 and NO, whose production is mediated by COX-2 and iNOS, were also significantly inhibited by Au_25_Sv_9_ treatment (Figure 3g,h).

### 3.4. Au_25_Sv_9_ Inhibits Activation of NF-κB Signaling Pathway in LPS-Stimulated HBZY-1 Cells

**Figure 4:**
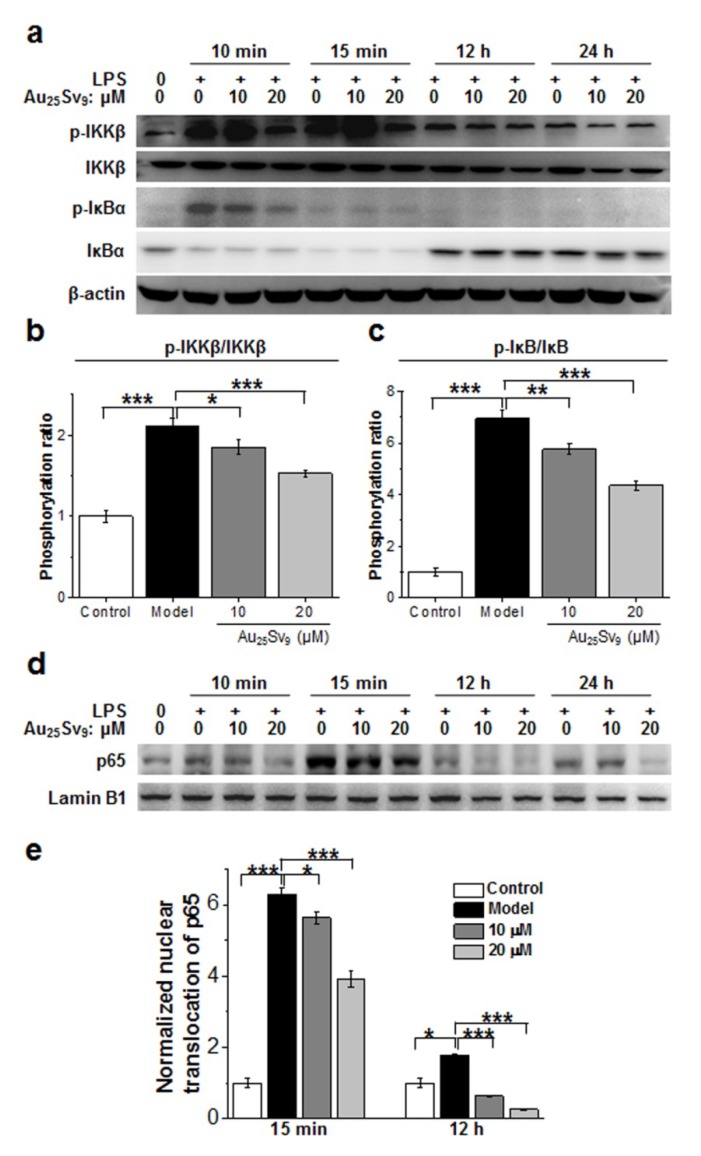
The inhibitory effect of Au_25_Sv_9_ on NF-κB (nuclear factor kappa B) pathway activation in LPS (lipopolysaccharide)-stimulated HBZY-1 cells. (**a**) Western blotting detection of the expression and phosphorylation of IKKβ (IκB kinase beta) and IκBα (NF-κB inhibitor alpha) in cytoplasm after incubation with LPS and Au_25_Sv_9_ for different times. β-actin was used as the loading control. (**b**) The relative phosphorylation ratio of IKKβ (p-IKKβ/ IKKβ) at 10 min was densitometry-analyzed. Data was expressed as the mean ± standard deviation (n = 3). (**c**) The relative phosphorylation ratio of IκBα (p-IκBα/ IκBα) at 10 min was densitometry-analyzed. Data was expressed as the mean ± standard deviation (n = 3). (**d**) Western blotting detection of NF-κB (p65) in the nucleus of HBZY-1 cells after incubation with LPS and Au_25_Sv_9_ for different times. Lamin B1 was used as the loading control. (**e**) The relative nuclear translocation of p65 at 15 min was densitometry-analyzed. Lamin B1 was used to normalize the data. Data was expressed as the mean ± standard deviation (n = 3). * P < 0.05, ** P < 0.01, *** P < 0.001.

NF-κB is a key transcription factor that regulates a variety of genes participating in inflammatory responses, including IL-6, TNF-α, iNOS and COX-2 [22,23]. Moreover, the NF-κB pathway is dramatically activated in the organs of septic animal models and patients, associated with the pathogenesis of septic AKI [24]. Therefore, we evaluated the effects of Au_25_Sv_9_ on LPS-induced NF-κB activation in HBZY-1 cells. After treatment with Au_25_Sv_9_ (10 μM or 20 μM) and LPS, the activation of the NF-κB signaling pathway in the HBZY-1 cell was detected by western blotting at different time points (10 min, 15 min, 12 h and 24 h). The results showed that the phosphorylation of IκBα and IKKβ was dramatically induced within 10 min. Treatment with Au_25_Sv_9_ can effectively suppress this activation in a dose-dependent manner at several time points (Figure 4a–c). In addition, the nuclear translocation of NF-κB (p65) was significantly enhanced at 15 min after LPS stimulation, while it was suppressed instantly by Au_25_Sv_9_ in a dose-dependent manner (Figure 4d,e).

### 3.5. Effects of Au_29_SG_27_ Gold Clusters on LPS-Stimulated HBZY-1 Cells

**Figure 5:**
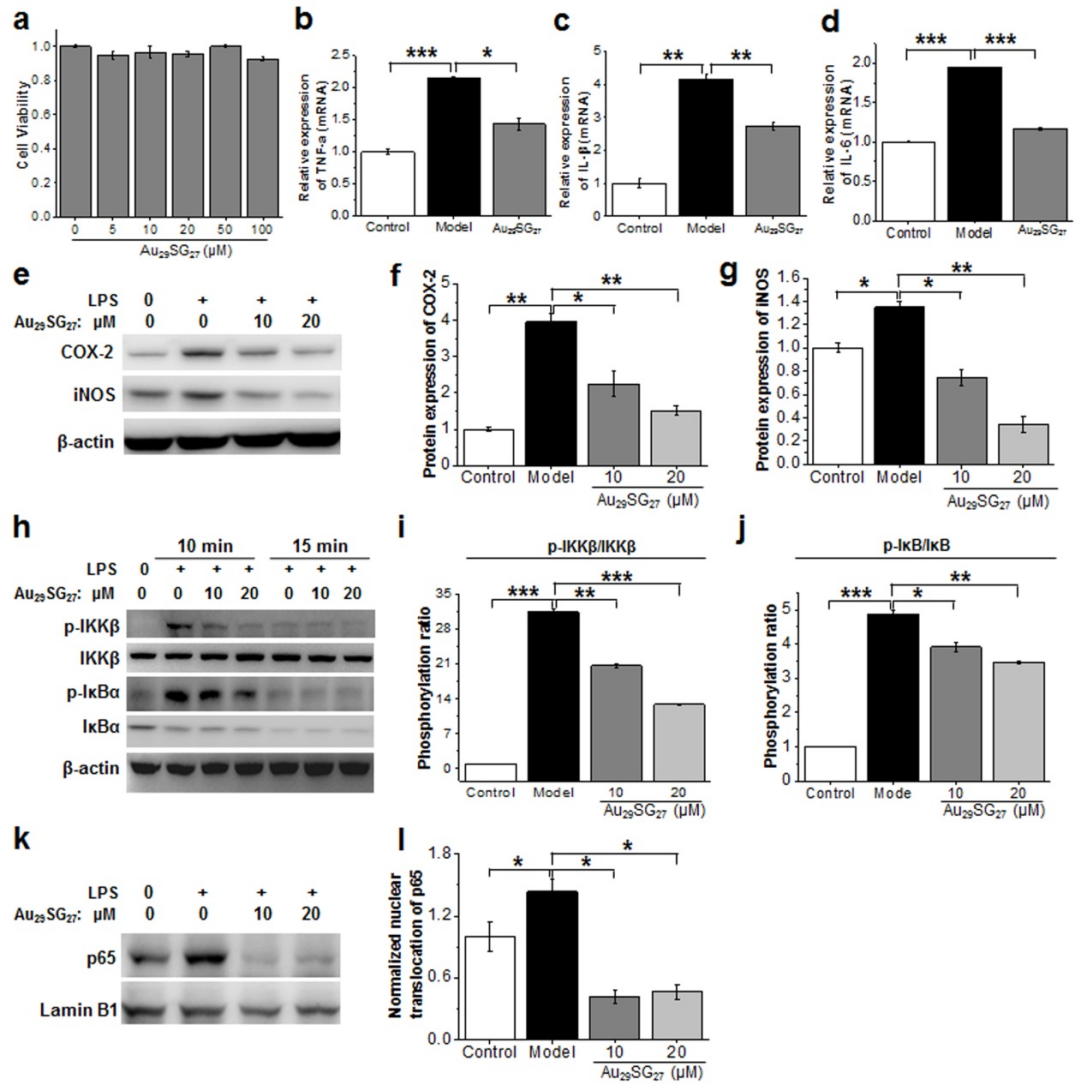
The effect of Au_29_SG_27_ on LPS (lipopolysaccharide)-induced inflammation and NF-κB (nuclear factor kappa B) activation in HBZY-1 cells. (**a**) Cell viability of HBZY-1 cells after incubation with different doses of Au_29_SG_27_ for 24 h. Data was presented as the mean of triplicate experiments. (**b**–**d**) The effects of Au_29_SG_27_ on the LPS-induced transcription of TNF-α (tumor necrosis factor alpha), IL-1β (interleukin-1 beta) and IL-6 (interleukin-6) in HBZY-1 cells after 12 h incubation were detected by RT-qPCR (real-time quantitative PCR). GAPDH was used to normalize the data. Results were expressed as the mean of three repeated experiments ± standard deviation, * P < 0.05, ** P < 0.01, *** P < 0.001. (**e**–**g**) Western blotting detection (**e**) and densitometry analysis (**f**,**g**) of the expression of COX-2 (Cyclooxygenase-2) and iNOS (inducible nitric oxide synthase) at 12 h. β-actin was used as the internal control and was used to normalize the data. Data was presented as the mean of triplicate experiments. (**h**) Western blotting detection of the expression and phosphorylation of IKKβ (IκB kinase beta) and IκBα (NF-κB inhibitor alpha) in cytoplasm after incubation with LPS and Au_29_SG_27_ for different times. β-actin was used as the loading control. (**i**) The relative phosphorylation ratio of IKKβ (p- IKKβ/ IKKβ) at 10 min was densitometry-analyzed. Data was expressed as the mean ± standard deviation (n = 3). (**j**) The relative phosphorylation ratio of IκBα (p-IκBα/ IκBα) at 10 min was densitometry-analyzed. Data was presented as the mean ± standard deviation (n = 3). (**k**) Western blotting detection of NF-κB (p65) in the nucleus of HBZY-1 cells after incubation with LPS and Au_29_SG_27_ for 12 h. Lamin B1 was used as the loading control. (**l**) The relative nuclear translocation of p65 was densitometry-analyzed. Lamin B1 was used to normalize the data. Data was presented as the mean ± standard deviation (n = 3). * P < 0.05, ** P < 0.01, *** P < 0.001.

To clarify whether the anti-inflammation activity in LPS-stimulated HBZY-1 cells is attributed to the intrinsic bioactivity of gold clusters rather than that of peptides, another peptide-templated gold cluster (Au_29_SG_27_) was used to evaluate its activity in the same model. Similar to Au_25_Sv_9,_ the Au_29_SG_27_ gold clusters did not induce any obvious cytotoxicity in HBZY-1 cells either, even at high concentrations (Figure 5a). The results showed that the LPS-induced transcriptions of IL-6, IL-1β and TNF-α were all significantly suppressed by 20 μM Au_29_SG_27_ (Figure 5b–d). LPS-induced activation of the NF-κB pathway and the subsequent overexpression of COX-2 and iNOS were effectively inhibited by Au_29_SG_27_ pretreatment (Figure 5e–l). 

## 4. Discussion

Sepsis, a dominant cause of death in ICU, is most likely to damage kidneys [6]. Acute kidney injury (AKI) is a common complication of sepsis, with an incidence rate of up to 50% in patients with severe sepsis, and the mortality rate can rise up to 70% [9]. Therefore, ameliorating septic AKI promptly and effectively would be helpful to improve the prognosis of sepsis. Despite some targeted treatments for specific inflammatory factors having been explored, little improvement has been shown regarding the prognosis of septic AKI, and the mortality rate remains very high [25]. Therefore, it is still crucial to develop new treatment strategies for sepsis-induced AKI. 

To the best of our knowledge, sepsis-caused acute kidney injury is mainly due to overwhelming and continuous inflammatory response [1,26]. In previous studies, we found that peptide-templated gold clusters possessed a wide anti-inflammatory activity in mouse macrophages and microglia cells via suppressing some pro-inflammatory factors, including IL-6, IL-1β, TNF-α, COX-2 and iNOS [13,15,16]. Considering the key role of these inflammatory factors in the pathogenesis of septic AKI [20,21,27], we speculate that gold clusters may play a similar anti-inflammatory role in this disease model. Therefore, this study was conducted to evaluate the potential effects of gold clusters on septic AKI in vitro. In the glomerulus, mesangial cells occupy a central position and participate in the pathogenesis of AKI [28]. LPS is usually used to induce septic AKI in animal models [29,30]. Therefore, to investigate the effects of gold clusters on septic AKI in vitro, the model of the LPS-stimulated rat mesangial cell HBZY-1 was used in this study.

An ultra-small size and well-defined molecular structure make peptide-templated gold clusters possess various unique physicochemical properties that are not seen in the corresponding bigger nanomaterials [11,12]. In particular, their excellent biocompatibility and intrinsic biomedical properties have attracted extensive attention in recent years [11,12]. In this study, the viability of HBZY-1 cells was not affected by both gold clusters treatments, even at a high dose of 100 μM, indicating their good biocompatibility. IL-6, IL-1β and TNF-α are three key pro-inflammatory factors involved in the development of septic AKI [27]. COX-2 plays a key role in renal disease. The selective inhibition of iNOS is an effective method for the treatment of septic AKI [21,31]. These factors were all significantly up-regulated in LPS stimulated HBZY-1 cells, suggesting that the in vitro septic AKI model was successfully established. Gold clusters reduced the LPS-induced overexpression of these factors effectively, indicating their potential anti-inflammatory effects on septic AKI. NF-κB is a key transcription factor that regulates these pro-inflammatory genes participating in the inflammatory responses of various biological processes, including AKI [32]. According to reports, the NF-κB pathway was dramatically activated in the organs of septic animal models and patients [24]. In addition, the inhibition of NF-κB-mediated inflammation has been demonstrated to exhibit a protective effect against sepsis in various organs [33]. Therefore, we investigated the effects of gold clusters on the NF-κB signaling pathway in LPS-stimulated HBZY-1 cells. In unstimulated cells, NF-κB is sequestered in the cytoplasm through interaction with inhibitory proteins IκB. LPS stimulation will induce the phosphorylation and subsequent degradation of IκB proteins, and the released NF-κB enters the nucleus to induce the expression of specific target genes. Our results showed that gold clusters significantly suppressed the LPS-induced phosphorylation of IKKβ and IκBα and p-65 nuclear translocation. These data suggest that the gold clusters inhibit renal inflammation, probably by preventing NF-κB activation. 

In this study, two types of gold clusters prepared with different peptides showed similar anti-inflammatory and NF-κB-suppressing activities in LPS-stimulated HBZY-1 cells, suggesting that the biomedical activity of gold clusters mainly depends on the intrinsic biochemical properties, rather than on those of the ligand peptide. Whether they can exert such an inflammation activity effectively in animal models and even in the human body needs further experimental verification. On the other hand, previous studies have demonstrated that the ultra-small size of gold clusters allows them to penetrate kidney tissue to decrease in vivo toxicity by renal clearance [13,34,35,36]. This objectively results in a targeted accumulation of gold clusters in the kidney, which is beneficial to them exerting their anti-inflammatory effects within the kidney in vivo and which facilitates the clinical transformation for the treatment of septic AKI in the future. However, this conjecture still needs to be further verified in AKI animal models. In addition, the biosafety and pharmacokinetic characteristics of gold clusters in vivo need to be evaluated comprehensively in animal models. The clinical transformation could only be promoted by completing these preclinical systematic studies.

## 5. Conclusions

In summary, this study demonstrates that gold clusters can mitigate LPS-induced inflammatory responses in rat mesangial cells via inhibiting the expression of pro-inflammatory factors, including IL-6, IL-1β, TNF-α, COX-2 and iNOS. This inhibitory effect of gold clusters on LPS-stimulated pro-inflammatory expression probably relies on the suppression of the NF-κB pathway. These results suggest the therapeutic potential of gold clusters on septic AKI. 

## Figures and Tables

**Table 1:** The primers sequences for PCR detection.

Gene	Forward	Reverse
COX-2	ACTTACAGGAGAGAAGGAAATGGCT	GTATTGAGGAGAACAGATGGGAT
iNOS	AGCCGCACCACCCTCCTTGT	GCCTCTTGTCTTTGACCCAGTAG
TNF-α	CTCAAGCCCTGGTATGAGCC	GGCTGGGTAGAGAACGGATG
IL-1β	GGAAGGCAGTGTCACTCATTGTG	GGTCCTCATCCTGGAAGCTCC
IL-6	TCCTACCCCAACTTCCAATGC	TAGCACACTAGGTTTGCCGAG
GAPDH	AGACAGCCGCATCTTCTTGT	CTTGCCGTGGGTAGAGTCAT
